# SRI-30827, a novel allosteric modulator of the dopamine transporter, alleviates HIV-1 Tat-induced potentiation of cocaine conditioned place preference in mice

**DOI:** 10.1515/nipt-2023-0022

**Published:** 2023-12-01

**Authors:** Haylee R. Hammond, Shainnel O. Eans, Thomas J. Cirino, Subramaniam Ananthan, Ana Catya Jimenez-Torres, Jun Zhu, Jay P. McLaughlin

**Affiliations:** Department of Pharmacodynamics, College of Pharmacy, University of Florida, Gainesville, FL 32610, USA; Department of Pharmacodynamics, College of Pharmacy, University of Florida, Gainesville, FL 32610, USA; Department of Pharmacodynamics, College of Pharmacy, University of Florida, Gainesville, FL 32610, USA; Department of Chemistry, Drug Discovery Division, Southern Research Institute, Birmingham, AL 35205, USA; Department of Drug Discovery and Biomedical Sciences, College of Pharmacy, University of South Carolina, Columbia, SC 29208, USA; Department of Drug Discovery and Biomedical Sciences, College of Pharmacy, University of South Carolina, Columbia, SC 29208, USA; Department of Pharmacodynamics, College of Pharmacy, University of Florida, 1345 Center Drive, Building JHMHC, P.O. Box 100487, Gainesville, FL 32610, USA

**Keywords:** HIV-1 Tat protein, dopamine transporter, allosteric modulator, cocaine, conditioned place preference, drug abuse

## Abstract

**Objectives:**

HIV-1 Tat (transactivator of transcription) protein disrupts dopaminergic transmission and potentiates the rewarding effects of cocaine. Allosteric modulators of the dopamine transporter (DAT) have been shown to reverse Tat-induced DAT dysfunction. We hypothesized that a novel DAT allosteric modulator, SRI-30827, would counteract Tat-induced potentiation of cocaine reward.

**Methods:**

Doxycycline (Dox)-inducible Tat transgenic (iTat-tg) mice and their G-tg (Tat-null) counterparts were tested in a cocaine conditioned place preference (CPP) paradigm. Mice were treated 14 days with saline, or Dox (100 mg/kg/day, i.p.) to induce Tat protein. Upon induction, mice were place conditioned two days with cocaine (10 mg/kg/day) after a 1-h daily intracerebroventricular (i.c.v.) pretreatment with SRI-30827 (1 nmol) or a vehicle control, and final place preference assessed as a measure of cocaine reward.

**Results:**

Dox-treatment significantly potentiated cocaine-CPP in iTat-tg mice over the response of saline-treated control littermates. SRI-30827 treatment eliminated Tat-induced potentiation without altering normal cocaine-CPP in saline-treated mice. Likewise, SRI-30827 did not alter cocaine-CPP in both saline- and Dox-treated G-tg mice incapable of expressing Tat protein.

**Conclusions:**

These findings add to a growing body of evidence that allosteric modulation of DAT could provide a promising therapeutic intervention for patients with comorbid HIV-1 and cocaine use disorder (CUD).

## Introduction

While severe HIV-associated neurocognitive disease (HAND) has declined significantly, milder impairments in attention, concentration, memory, and motivation persist, affecting approximately 50 % of HIV-positive patients [1]. Moreover, substance use disorders are commonly comorbid with HIV infection and are known to exacerbate the progression of HAND [2]. As HIV is not thought to directly infect neurons, the dysregulation of motivational processes has been attributed to the action of HIV-1 proteins [3], some of which have been directly linked to cognitive impairment and brain-injury. Among these proteins, transactivation of transcription (Tat), is known to act as a negative allosteric modulator of the dopamine transporter (DAT), inhibiting dopamine (DA) uptake [4, 5]. Our recent study demonstrates that the disruption of DAT-mediated dopaminergic transmission caused by Tat contributes to Tat-induced potentiation of cocaine reward and deficits in learning and memory seen in HAND [5], making Tat an attractive pharmacologic target [6]. Our previous study reported that SRI-30827, a closely related analog of SRI-32743, attenuated Tat-induced inhibition of [^3^H]WIN35428 binding through its influence on tyrosine470 and tyrosine88 residues in the EL6 region of hDAT [7]. These two hDAT residues are critical for Tat protein’s allosteric modulation of DAT [4]. Furthermore, SRI-32743 dose-dependently reversed Tat-induced potentiation of cocaine-CPP and impairment of novel object recognition (NOR) in mice [6]. The doses of SRI-32743 tested were without effect on cocaine-CPP or NOR in mice lacking Tat protein expression. Extending the SRI-32743 results, this study further examined whether SRI-30827 with a quinazoline structure may attenuate Tat-induced potentiation of cocaine-CPP.

## Materials and methods

### Transgenic mouse models

Adult male inducible Tat transgenic (iTat-tg) mice and G-tg (Tat-null) mice [8] were obtained from colonies at the University of Florida as reported previously [6]. Both the iTat-tg and Tat-null mice genetically expresses a “tetracycline-on (TETON)” system, but only iTat-tg mice possess the Tat_1–86_ coding gene [8]. Integration into the gene regulator for the astrocyte-specific glial fibrillary acidic protein (GFAP) promoter confines Tat expression to the CNS [8, 9]. Based on the previously demonstrated expression of Tat protein [8, 9], the current study utilized doxycycline at a 100 mg/kg/day dose, i.p., for 14 days to maximize induction of Tat protein.

### Drugs

All drugs injected i.p. or s.c. were administered in a volume of 10 mL/kg of body weight. Drugs injected i.c.v. were administered in a fixed volume of 5 μL. Cocaine hydrochloride and doxycycline hyclate (Sigma-Aldrich, St. Louis, MO, USA) were dissolved in saline (0.9 % sodium chloride). SRI-30827 synthesized at the Southern Research Institute (Birmingham, AL, USA) [7] is poorly soluble in saline, and was therefore dissolved in 100 % DMSO and administered at 1 nmol/day, i.c.v.

### Conditioned place preference (CPP)

**Figure 1:**
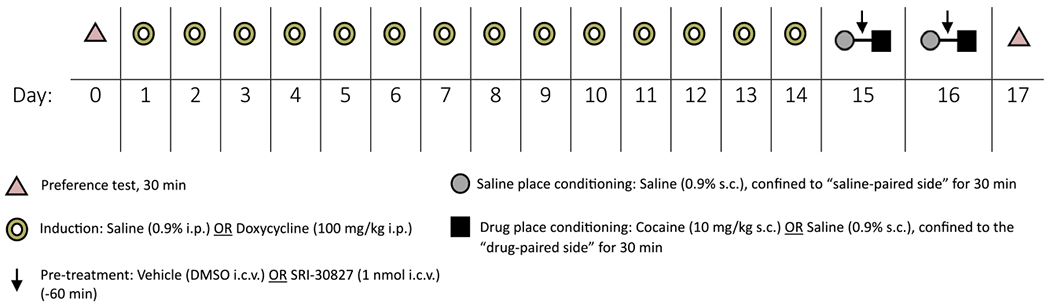
CPP experimental design schematic. An initial, pre-conditioning preference was determined by measuring the amount of time the individual mice spent in each chamber during a 30-min testing period. Mice were then treated with saline (0.9 %) or Dox (100 mg/kg) via i.p. injection for 14 days before the start of place-conditioning. On days 15 and 16, mice were given saline (0.9 %) s.c. and consistently confined to a randomly assigned outer compartment, with half of each group in the right chambers and half in the left. Three hours later, mice were given a pre-treatment of either SRI-30827 (1 nmol) or vehicle (DMSO) via i.c.v injection. An hour after this pre-treatment, mice underwent drug-conditioning (with either saline or cocaine 10 mg/kg s.c.) and were confined to the opposite, “drug-paired” compartment for 30 min. Twenty-four hours after the completion of their two-day conditioning cycle, mice were tested for post-conditioning place preference by allowing them access to all compartments and measuring the time they spent in each chamber over a 30-min testing period.

Cocaine-CPP was performed with a three-chamber apparatus (San Diego Instruments, San Diego, CA, USA) using a counterbalanced design [6]. Place conditioning was performed on days 15 and 16 as reported [6], described in Figure 1. On test days, mice were allowed to move freely between chambers in a 30-min preference test.

## Results

### iTat-tg mice

#### Exposure to Tat protein causes potentiation of cocaine-CPP

Prior to place conditioning, there were no significant differences in the initial place preference responses between any of the six groups of iTat-tg mice (one-way ANOVA: *F*_(5,156)_=0.22, *p*=0.95). All groups of iTat-tg mice conditioned with cocaine demonstrated CPP (Table 1), while, as expected, mice conditioned with saline did not (Table 1).

**Figure 2:**
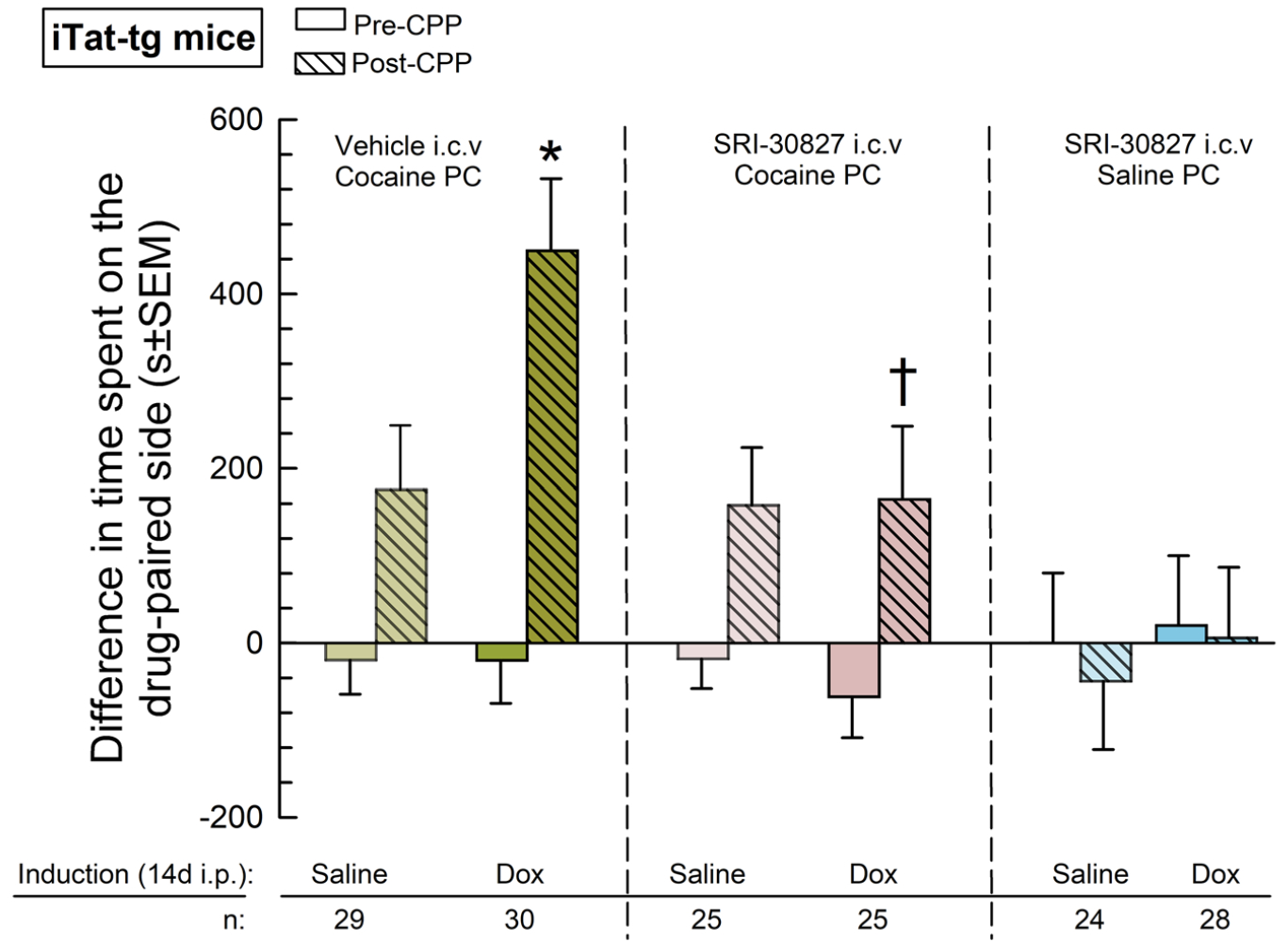
iTat-tg cocaine and saline conditioned place preference. An initial, pre-conditioning preference was determined by measuring the amount of time the individual mice spent in each chamber during a 30-min testing period. Mice were then treated with saline (0.9 %) or Dox (100 mg/kg) via i.p. injection for 14 days before the start of place-conditioning. On days 15 and 16 (see Figure 1), mice were given saline (0.9 %) s.c. and confined to a randomly assigned outer compartment. Three hours later, mice were given a pre-treatment of either SRI-30827 (1 nmol) or vehicle (DMSO) via i.c.v injection. An hour after this pre-treatment, mice underwent drug-conditioning (with either saline or cocaine 10 mg/kg s.c.) and confined to the opposite, “drug-paired” compartment for 30 min. Twenty-four hours after the completion of their two-day conditioning cycle, mice were tested for post-conditioning preference by allowing them access to all compartments and measuring the time they spent in each chamber over a 30-min testing period, with the difference in time spent reported in seconds ± standard error of the mean (SEM). Number of mice in each treatment group is as listed in the figure. *p<0.05 versus matching saline-treated post-conditioning response; ^†^*p*<0.05 versus post-conditioning response of Dox-treated mice administered i.c.v. vehicle prior to cocaine place conditioning.

When pretreated with vehicle (i.c.v.) and place conditioned with cocaine, control iTat-tg mice that received a 14-day induction with doxycycline demonstrated a significant place preference (factor: treatment × conditioning, *F*_(5,155)_=3.90, *p*=0.002; two-way RM ANOVA with Tukey’s HSD post hoc test, Figure 2) that was 2.6-fold significantly greater when compared to their saline-induced littermates (Figure 2, Left panel; *p=0.04, Tukey’s HSD).

#### SRI-30827 ameliorates Tat-induced potentiation of cocaine-CPP

SRI-30827 pretreatment significantly reduced cocaine-CPP in doxycycline-induced iTat-tg mice compared to those pretreated with vehicle (Figure 2, left and center panels; ^†^*p*=0.04, Tukey’s HSD). There was no difference in cocaine-CPP between doxycycline-induced and saline-induced iTat-tg mice pretreated with SRI-30827 (Figure 2, center panel; *p*=0.35, Tukey’s HSD).

#### SRI-30827 does not affect cocaine-CPP in iTat mice induced with saline

Among the control mice given 14 days of saline and place conditioned with cocaine, there was no difference in cocaine-CPP between groups pretreated with SRI-30827 or vehicle (Figure 2, left and central panels; *p*>0.99, Tukey HSD), demonstrating that SRI-30827 itself does not alter cocaine-CPP in the absence of Tat protein.

#### SRI-30827 itself does not demonstrate rewarding or adverse effects

Mice pretreated with SRI-30827 and place conditioned with only saline did not show a place preference for either the saline-alone or SRI-30827 + saline chamber (Figure 2, right panel; *p*=0.996, Tukey’s HSD).

### Tat-null mice

#### SRI-30827 does not alter cocaine-CPP in Tat-null mice incapable of expressing Tat protein

**Figure 3:**
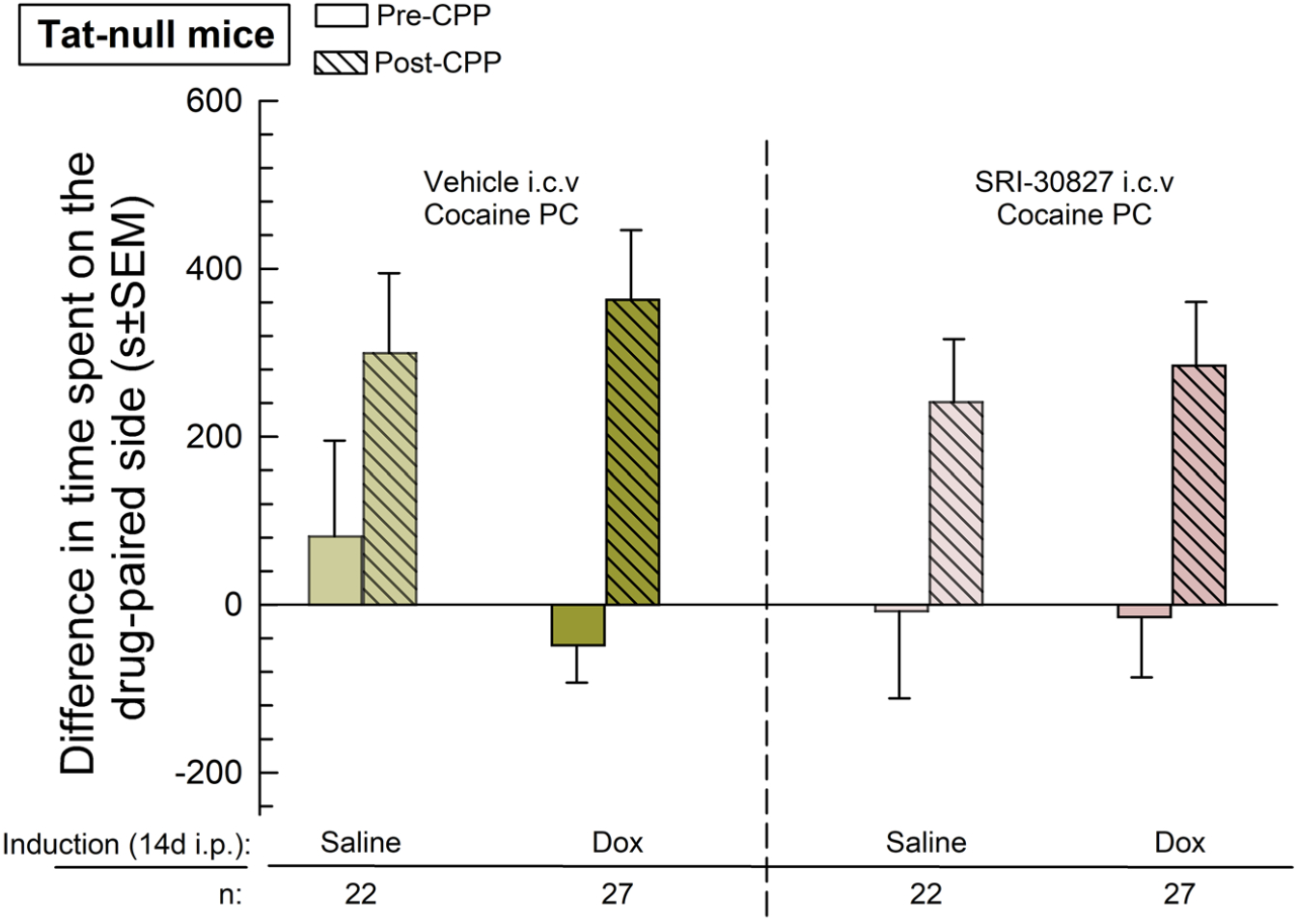
Tat-null cocaine conditioned place preference. An initial, pre-conditioning preference was determined by measuring the amount of time the individual mice spent in each chamber during a 30-min testing period. Mice were then treated with saline (0.9 %) or Dox (100 mg/kg) via i.p. injection for 14 days before the start of place-conditioning. On days 15 and 16 (see Figure 1), mice were given saline (0.9 %) s.c. and confined to a randomly assigned outer compartment. Three hours later, mice were given a pre-treatment of either SRI-30827 (1 nmol) or vehicle (DMSO) via i.c.v injection. An hour after this pre-treatment, mice underwent drug-conditioning (with cocaine 10 mg/kg s.c.) and confined to the opposite, “drug-paired” compartment for 30 min. Twenty-four hours after the completion of their two-day conditioning cycle, mice were tested for post-conditioning preference by allowing them access to all compartments and measuring the time they spent in each chamber over a 30-min testing period, with the difference in time spent reported in seconds ± standard error of the mean (SEM). Number of mice in each treatment group is as listed in the figure.

Prior to place conditioning, there were no significant differences between the initial place preference responses between any of the four Tat-null groups (one-way ANOVA: *F*_(3,94)_=0.43, *p*=0.73). Tat-null mice demonstrated a significant cocaine-CPP both globally (two-way RM ANOVA: F_(1,94)_=32.5, *p*<0.0001) and in their respective groups (Table 1), but there was no significant difference in cocaine-CPP response across groups regardless of induction with doxycycline and/or pre-treatment with SRI-30827 (Two-way RM ANOVA, *F*_(3,94)_=0.22, *p*=0.88). This suggests that in the absence of Tat protein, this dose of SRI-30827 was without direct effect on cocaine-CPP (Figure 3).

## Discussion and conclusions

Although both Tat and cocaine interact with DAT, cocaine competitively blocks the DAT uptake site, whereas Tat interacts with DAT in a allosteric modulatory manner [5]. Together they produce synergistic dysfunction of dopaminergic transmission, thought to contribute to the progression of HAND and altered drug reward [6]. Consistent with these findings, 14 day Dox-treated iTat-tg mice displayed a 2.6-fold potentiation of cocaine-CPP compared to the response of vehicle-treated control littermates. Potentiation of cocaine-CPP was not observed in saline-treated iTat-tg or Dox-treated Tat-null mice, implicating the role of Tat in the potentiated drug reward, consistent with earlier reports [6]. Treatment with the novel allosteric modulator of the DAT, SRI-30827, reversed Tat-induced potentiation of cocaine-CPP in Dox-treated iTat-tg mice, but had no effect on the magnitude of cocaine-CPP in control animals. These findings extend previous *in vitro* DAT binding and functional assay data with SRI-30827 [7], and are consistent with results observed with the related allosteric modulator, SRI-32743 [6]. Collectively, this work adds to a growing body of evidence that allosteric modulation of DAT may provide a promising therapeutic intervention for patients with comorbid HIV-1 and CUD.

The iTat-tg mouse model has been instrumental in clarifying the CNS and behavioral effects of Tat alone, suggesting exposure to HIV-1 Tat protein is sufficient to induce CNS dysfunction. However, other viral components presently unexamined may contribute to HAND and the potentiation of drug reward in patients with HIV-1. Notably, mice infected with Eco-HIV, an engineered virus with 9 of 11 HIV regulatory proteins, exhibit elevated stress-induced reinstatement of cocaine seeking behavior [10] and blunted extinction of cocaine preference [11]. Future testing of allosteric modulators in these models may elucidate the contribution of Tat to increased drug reward when in the presence of other concomitant viral proteins, and further characterize the therapeutic potential of allosteric modulators of the DAT.

A limitation of the current study lies in the poor solubility and brain penetration of SRI-30827. Circumventing these limitations, the current study utilized DMSO and i.c.v. administration under conditions known not to negatively impact place preference behavior [12]. However, structural modification of the SRI-allosteric modulators to improve their solubility and penetrance of the BBB would be expected to increase their clinical value. Confirming this are earlier reports where the more-druggable SRI-32743 produced similar effects as SRI-30827 did presently, but after systemic administration [6]. These compounds only ameliorate Tat-mediated DAT dysfunction. Alternatively, inhibiting Tat protein itself might slow viral replication and the production of viral proteins, potentially ameliorating other off-target effects. Molecular modeling, computational screening, and some *in vitro* analyses have identified several candidate compounds termed “Tat antagonists” [13]. The natural product Didehydro-Cortistatin A (dCA) was found to prevent Tat-induced potentiation of cocaine-CPP in iTat-tg mice [12], and although Tat antagonist Ro 24-7429 failed to show measurable effects even at high doses in Phase I clinical trials [14], work continues to develop Tat antagonists as therapeutics to eliminate the activities of Tat protein. Thus, these results demonstrate that DAT allosteric modulators like SRI-30827 and SRI-32743 which attenuate cocaine- and Tat-binding to DAT may provide an early effective intervention for symptomatic relief from neurocognitive deficits along with substance abuse in the early stage of HIV-infected individuals.

## Figures and Tables

**Table 1:** iTat-tg and Tat-null conditioned place preference results. A series of two-tailed Student’s t-tests (adjusted for multiple comparisons) were performed for each group to determine if the mice displayed a difference in their place preference as a result of conditioning. Both initial (pre-CPP) and final (post-CPP) values for each group are reported as the mean difference in time spent (in seconds) in the drug-paired compartment. The difference in time spent in the drug-paired compartment was calculated by simply subtracting the amount of time spent in the saline-paired compartment from the amount of time spent in the drug-paired compartment over the 30-min testing period. Variability in this value is reported as standard error of the mean (SEM), in seconds (s). As expected, groups place conditioned with cocaine produced a significant place preference for the drug-paired compartment, while groups conditioned only with saline did not show place preference. Groups are labelled as: induction (saline 14d or Dox 14d), pre-treatment (vehicle or SRI-30827), and drug place conditioning (cocaine PC or saline PC).

	n	Mean of pre-CPP, s	Mean of post-CPP, s	Difference, s	SEM of difference, s	q-value
*iTat mice*						
Dox 14d, vehicle, cocaine PC	30	−19.77	449.6	−469.4	84.24	0.000026
Dox 14d, SRI-30827, cocaine PC	25	−61.56	165.0	−226.6	86.18	0.031166
Saline 14d, vehicle, cocaine PC	29	−19.31	175.9	−195.2	78.06	0.031166
Saline 14d, SRI-30827, cocaine PC	25	−18.08	158.1	−176.2	77.22	0.040028
Dox 14d, SRI-30827, saline PC	28	20.21	5.929	14.29	111.5	0.756682
Saline 14d, SRI-30827, saline PC	24	0.2500	−43.67	43.92	133.4	0.752397
*Tat-null mice*						
Dox 14d, vehicle, cocaine PC	27	−48.26	362.9	−411.2	92.78	0.000456
Dox 14d, SRI-30827, cocaine PC	27	−14.42	284.5	−298.9	122.1	0.021630
Saline 14d, vehicle, cocaine PC	22	81.82	299.6	−217.7	93.84	0.023084
Saline 14d, SRI-30827, cocaine PC	22	−7.609	240.9	−248.5	89.41	0.017007

## Data Availability

Not applicable.
